# Integrated Analyses of mRNA and microRNA Regulatory Networks at Different Soil Moisture in Tropical Earthworm *Eudrilus eugeniae*

**DOI:** 10.3390/biology15161387

**Published:** 2026-08-13

**Authors:** Zhen Dong, Wai Lok So, Jacky Chi Ki Ngo, Hon-Ming Lam, Ting-Fung Chan, Jerome Ho Lam Hui

**Affiliations:** 1 State Key Laboratory of Agrobiotechnology, School of Life Sciences, The Chinese University of Hong Kong, Hong Kong SAR, China; 2 Institute of Environment, Energy and Sustainability, The Chinese University of Hong Kong, Hong Kong SAR, China; 3 Simon F.S. Li Marine Science Laboratory, The Chinese University of Hong Kong, Hong Kong SAR, China

**Keywords:** desiccation, earthworm, microRNA, moisture, sustainable agriculture, *Eudrilus eugeniae*

## Abstract

Earthworms play crucial roles in soil fertility and are vital to sustainable agriculture and environmental stability. Synchronously, they are highly sensitive to their environment, which limits their abundance and activities. Existing research primarily focuses on their protein-coding gene responses at different chemicals and/or temperature stresses. This study utilized the tropical earthworm *Eudrilus eugeniae* as a model to investigate how the expression of protein-coding genes and microRNAs are influenced in the anterior tissues at different moisture conditions (30%, 80%, and 100% water-holding capacity). We revealed that molecular chaperones sHsp20, Hsp70, a CHORD-containing protein, and HspBP1-like genes were differentially regulated, and Hsp70 family genes served as hub genes in this process. In addition, a novel lineage-specific microRNA, with an opposite expression trend, was predicted to target three chaperone genes (CHORD-containing protein, Hsp90-like, and Hsp40). Pull-down and dual-luciferase assays further verified their potential interactions. This study suggests holistic cooperation between transcriptional and post-transcriptional mechanisms in the anterior tissues of earthworms facing soil moisture variation and provides new insights into the effect of moisture on the adaptation of biological molecules in earthworms.

## Simple Summary

Earthworms are essential soil ecosystem engineers that contribute to nutrient cycling and agricultural production. Despite soil moisture being an important factor determining their survivorship, the molecular mechanisms underlying their responses at different soil moisture remain poorly understood. In this study, we examined the expression level changes in protein-coding genes and microRNAs in the tropical earthworm *Eudrilus eugeniae* when they were exposed to different soil moisture conditions. Molecular chaperone genes, particularly those in the heat shock protein family were upregulated under dry conditions, with Hsp70 members acting as hub genes. In addition, a lineage-specific microRNA was identified to target multiple chaperone genes. This is the first post-transcriptional study of responses in earthworm anterior tissues adapting to different moisture conditions.

## 1. Introduction

Global climate change is having effects on terrestrial ecosystems worldwide. Earthworms are one of the most crucial terrestrial ecosystem engineers which shape soil structure, enhance aeration and drainage, and accelerate nutrient cycling [1]. For human society, it was estimated that earthworms increase the primary productivity in crop yields [2] and contribute to around 6.5% of global cereal grain production [3]. Given earthworms breathe through their moist skin for gaseous exchange, they generally thrive in moist soil [4,5]. Climate change can alter both temperature and precipitation regimes, and climate extremes such as drought and flooding can have deleterious effects on earthworm populations [6]. Although most contemporary molecular studies have predominantly focused on the effects of temperature and chemical stressors on the expression of their protein-coding genes [7], much less is known about the effects of soil moisture.

Ecological and physiological studies have established that drought curtails earthworms’ activities and performance. These include burrowing cessation, loss of body mass, reduction in reproduction, and in some cases entering dormancy such as aestivation or diapause [6,8], and cocoon survivorship under desiccation also varies [9]. In the limited transcriptomic studies on these processes, desiccation suppressed the expression of lipid metabolism-related genes in *Eiseniella tetraedra* [4], and induced broad metabolic downregulation coupled with activation of oxidoreductase and DNA-repair pathway genes in *Carpetania matritensis* [8]. 

MicroRNAs are 21–23 nucleotides of noncoding RNAs that regulate gene expression at post-transcriptional level in animals [10]. As individual microRNAs can regulate multiple targets, they have also been proposed to coordinate rapid gene expression changes in the presence of environmental stresses [11,12]. In earthworms, research on microRNAs has been explored during tissue regeneration in *Perionyx excavatus* [13]. Nevertheless, the contribution of microRNA-gene regulatory network in earthworms at different soil moistures remains unknown.

The tropical earthworm *Eudrilus eugeniae* is a large epigeic species widely used in vermicomposting and as protein source for feeding animals. A draft genome resource has been established for this species [14]. In this study, we exposed *E. eugeniae* to different soil moisture conditions (30%, 80%, and 100% water-holding capacity (WHC)) and measured the responses of both protein-coding genes and microRNAs in anterior segments (1–6), aiming to understand the effect of soil moisture on the adaptation of microRNA:mRNA networks in earthworms for the first time.

## 2. Materials and Methods

### 2.1. Animal Culture, Species Identification, and Moisture Treatments

Earthworms *E. eugeniae* were purchased in June 2024 from a company (GreenFlow, Hong Kong SAR, China; https://www.greenflow.hk/). To confirm species identity, a small distal tail-tip tissue sample was excised from each individual for mitochondrial cytochrome c oxidase subunit I (COI) gene validation. The resulting wound healed during the subsequent 7-day starvation period; only individuals with complete wound closure and normal movement and burrowing behaviour were used. Species-confirmed individuals were cultured at 23 ± 1 °C at The Chinese University of Hong Kong and were starved for 7 days to ensure metabolic standardization before carrying out subsequent treatments. Five individuals of similar size were randomly chosen and assigned to each treatment group defined by soil water-holding capacity (WHC), including dry (30% WHC; D), control (80% WHC; C), and water-saturated (100% WHC; W) (Supplementary Figure S1). These percentages were chosen based on preliminary survivorship tests. The soil moisture levels were monitored daily using a calibrated moisture probe throughout the one-week exposure. After the treatment, the anterior segments (segments 1–6, encompassing the cerebral ganglia and ventral nerve cord) were dissected, frozen in liquid nitrogen and stored at −80 °C before further processing.

### 2.2. RNA Extraction and mRNA and sRNA Sequencing

Total RNA was isolated using TRIzol reagent (Invitrogen, Carlsbad, CA, USA) according to the manufacturer’s instructions. RNA quantification was performed using a Qubit 4 Fluorometer, and RNA purity was assessed via a NanoDrop One spectrophotometer (Thermo Fisher Scientific, Waltham, MA, USA). All five individuals per treatment were processed for RNA extraction; three high-quality samples were selected for paired mRNA-seq and small RNA-seq (*n* = 3 biological replicates per treatment), with both library types prepared from the same individuals (Supplementary Figure S1). RNA samples were sent to Novogene Co., Ltd. (Beijing, China) for library construction and sequencing. For each sample, two sequencing libraries were constructed including a poly(A)-enriched mRNA library for mRNA transcriptome profiling and a size-selected small RNA library for microRNA profiling. The mRNA and sRNA libraries were sequenced on an Illumina NovaSeq 6000 platform (Illumina Inc., San Diego, CA, USA) generating 150 bp paired-end reads and 50 bp single-end reads, respectively.

### 2.3. mRNA Transcriptome Assembly, Annotation, and Differential Expression Analysis

Raw sequencing reads were subjected to stringent quality control. Contaminant sequences (e.g., bacterial or viral DNA) were identified and removed using Kraken v2.0.8 [15] against the k2 standard database (v20240605). Quality filtering, including the removal of adapter sequences and low-quality bases, was performed using fastp v0.23.4 [16] to ensure high-quality clean data for downstream analyses. To construct a high-quality reference transcriptome, a dual-track assembly strategy was used to maximize transcript recovery, including: (i) a de novo assembly, which was generated using Trinity (v2.15.1) [17], and (ii) a genome-guided de novo assembly, which was constructed by aligning reads to the draft reference genome [14] via STAR (v2.7.10a) [18], followed by transcript reconstruction with StringTie (v2.1.1) [19]. The resulting assemblies were integrated into a unified consensus framework, and potential open reading frames (ORFs) were identified using TransDecoder (v5.5.0) (https://github.com/TransDecoder/TransDecoder) (accessed on 6 August 2026). CD-HIT (v4.8.1) [20] was then used to cluster sequences (similarity threshold of ≥95% and a minimum length of 300 bp), and transcript abundance was determined using Salmon (v1.10.0) [21]. From this refined dataset, the longest transcript and corresponding protein isoform at each genomic locus were extracted based on the GFF framework. The completeness and technical quality of the final assembled transcriptome were evaluated using BUSCO (Benchmarking Universal Single-Copy Orthologs) (v5.8.3) [22], and functional annotation was also performed using eggNOG-mapper (v2.1.12) [23] against the eggNOG database. Transcript abundance was quantified using the RSEM (RNA-Seq by Expectation-Maximization) pipeline (v1.3.3) [24], and a Bowtie2 index [25] was constructed from the final reference transcriptome. Differential expression analysis was conducted using the run_DE_analysis.pl edgeR statistical framework [26]. To account for varying sequencing depths, the raw count matrix was normalized using the Trimmed Mean of M-values (TMM) method [27]. Transcripts were filtered to retain only those exhibiting an expression level > 1 count per million (CPM) in at least two biological replicates [28]. Transcripts were defined as differentially expressed genes with Benjamini–Hochberg-adjusted *p* value (False Discovery Rate, FDR) < 0.05 and a minimum log_2_ fold change threshold of 1. 

### 2.4. Functional Enrichment Analyses

Gene Ontology (GO) and Kyoto Encyclopedia of Genes and Genomes (KEGG) pathway enrichment analyses were performed using the clusterProfiler (v4.4.4) package [29] in R (v4.3.3). Statistical significance was determined using a hypergeometric test, with *p* values adjusted for multiple testing (adjusted *p* < 0.05). All data visualizations, including heatmaps and volcano plots, were generated using the ggplot2 (v3.4.2) [30] and pheatmap (v1.0.13) packages [31].

### 2.5. Weighted Gene Co-Expression Network Analysis (WGCNA)

Weighted gene co-expression network analysis was performed using the R package WGCNA (v1.72) [32]. In brief, the expression matrix was pre-filtered to retain the top 5000 most variable genes based on median absolute deviation (MAD) across all samples. Hierarchical clustering was performed on all samples using average linkage and Euclidean distance. The pickSoftThreshold function was applied over a range of candidate powers, and a power of 16 was selected in this study based on the scale-free topology fit index and mean connectivity plot. An unsigned co-expression network was constructed using blockwiseModules function. Modules were identified via dynamic tree with a cut-off at minimum module size of 30 genes, and those with highly correlated eigengenes (Pearson correlation > 0.75) were subsequently merged. Each module was summarized by its eigengene, defined as the first principal component of the module’s expression profile across samples. Module eigengene values were compared using one-way analysis of variance (ANOVA) for each module. In addition, pairwise comparisons were conducted using Welch’s *t*-test. Modules with a *p* value < 0.05 were considered differentially expressed across groups. 

### 2.6. Quantitative Real-Time PCR (qRT-PCR) Validation

First-strand cDNA was synthesized from 1 μg of total RNA using the iScript Reverse Transcription Supermix (Bio-Rad, Hercules, CA, USA), and qRT-PCR reactions were carried out in 15 μL volumes using iTaq Universal SYBR Green Supermix (Bio-Rad) on a CFX96 Touch Real-Time PCR System (Bio-Rad, Hercules, CA, USA). Cycling conditions were shown as follows: an initial denaturation step at 95 °C for 3 min, followed by 39 cycles of 95 °C for 5 s, 57 °C for 30 s and 72 °C for 15 s. Primer amplification efficiencies were determined by generating standard curves with serial cDNA dilutions, and all the primer pairs used in this study had efficiencies > 90%. Relative transcript levels were calculated using the 2^−ΔΔCt^ method [33] and normalized to 3 housekeeping genes used in this study. All reactions were performed with two technical replicates and included eight biological replicates (two batches) per condition. These comprised the five individuals from the first batch and three additional individuals from an independent second batch to improve the reliability of the validation. Primer sequences are provided in Supplementary Table S1.

### 2.7. MicroRNA Annotation and Differential Expression Analysis

Raw sequencing reads were first subjected to adapter trimming using Cutadapt (v4.8) [34] and only reads with lengths between 18 and 45 nucleotides (nt) were retained. Redundant sequences were first collapsed using the FASTX-Toolkit (fastx_collapser) (v0.0.13), and a length filter of 18–27 nt was further applied to the collapsed reads using SeqKit (2.1.0) [35]. The processed small RNA tags were mapped to the draft *E. eugeniae* genome [14] using the mapper.pl module of the miRDeep2 (v2.0.13) [36] package. Retrieved hairpin sequences were searched against miRBase (v22) [37] using BLASTN (task: blastn-short, e-value < 1 × 10^−3^) (v2.15.0+) [38]. Candidates lacking sequence homology to those in miRBase were further investigated based on the MirGeneDB criteria (v3.0) [39]. The expression levels of identified microRNAs across all samples were quantified using the quantifier.pl module, allowing for two-nucleotide upstream/downstream shifts and a five-nucleotide window for mature sequence positioning. Raw counts were normalized, and differential expression analysis was performed using run_DE_analysis.pl edgeR statistical framework [26] integrated within the Trinity (v2.15.1) [17]. Significant differentially expressed microRNAs were defined with an FDR-adjusted *p* value < 0.05 and a minimum |log_2_ fold change| ≥ 1. Hierarchical clustering and principal component analysis (PCA) were conducted to evaluate the consistency of biological replicates and the global expression patterns across experimental conditions.

### 2.8. MicroRNA Target Gene Prediction

RNAhybrid (v2.1.2) [40] and miRanda (v3.3a) [41] were used to predict potential targets on the 3′ untranslated regions (3′UTRs) of the reference transcripts. To minimize false-positive predictions, the following filtering criteria were applied: (i) a statistical significance threshold of *p* < 0.05; (ii) a minimum free energy (MFE) of ΔG ≤ −20 kcal/mol; and (iii) a background model calibrated for 3′UTRs. Only interactions exhibiting sequence complementarity at the core seed region (positions 2–7) and/or the 3′ supplementary pairing region (positions 12–17) of the microRNAs were retained as potential targets [42].

### 2.9. Labelled microRNA Pull-Down Assay (LAMP)

Labelled microRNA pull-down assay was performed using Dynabeads™ M-280 Streptavidin (Cat. No. 11205D, Invitrogen, CA, USA) as previously described [43]. Total RNA was extracted from three biological replicates to test reproducibility. The pulled-down RNA was reverse-transcribed into cDNA as described above and amplified by PCR with gene-specific primers (Supplementary Table S2) using the following parameters: 95 °C for 5 min; 39 cycles of 95 °C for 30 s, 55 °C for 30 s, 72 °C for 20 s; and an additional step of 72 °C for 5 min. PCR products were resolved on 2.5% agarose gels and subjected to Sanger sequencing to confirm product specificity and identity. 

### 2.10. Dual-Luciferase Reporter Assay

A dual-luciferase reporter assay was used to validate potential interactions between microRNAs and target genes as previously described [43,44]. Primer information is shown in Supplementary Table S3. Transfection was performed in *Drosophila* S2 cells, and luciferase activity was measured 48 h post-transfection. Relative luciferase activity was calculated as the ratio of Renilla to firefly luminescence and normalized to the control group. To ensure reproducibility, the assay was performed across three independent biological replicates, each derived from a separately passaged batch of *Drosophila* S2 cells. 

## 3. Results

### 3.1. Differentially Expressed Genes in Anterior Tissues at Different Soil Moistures

**Figure 1:**
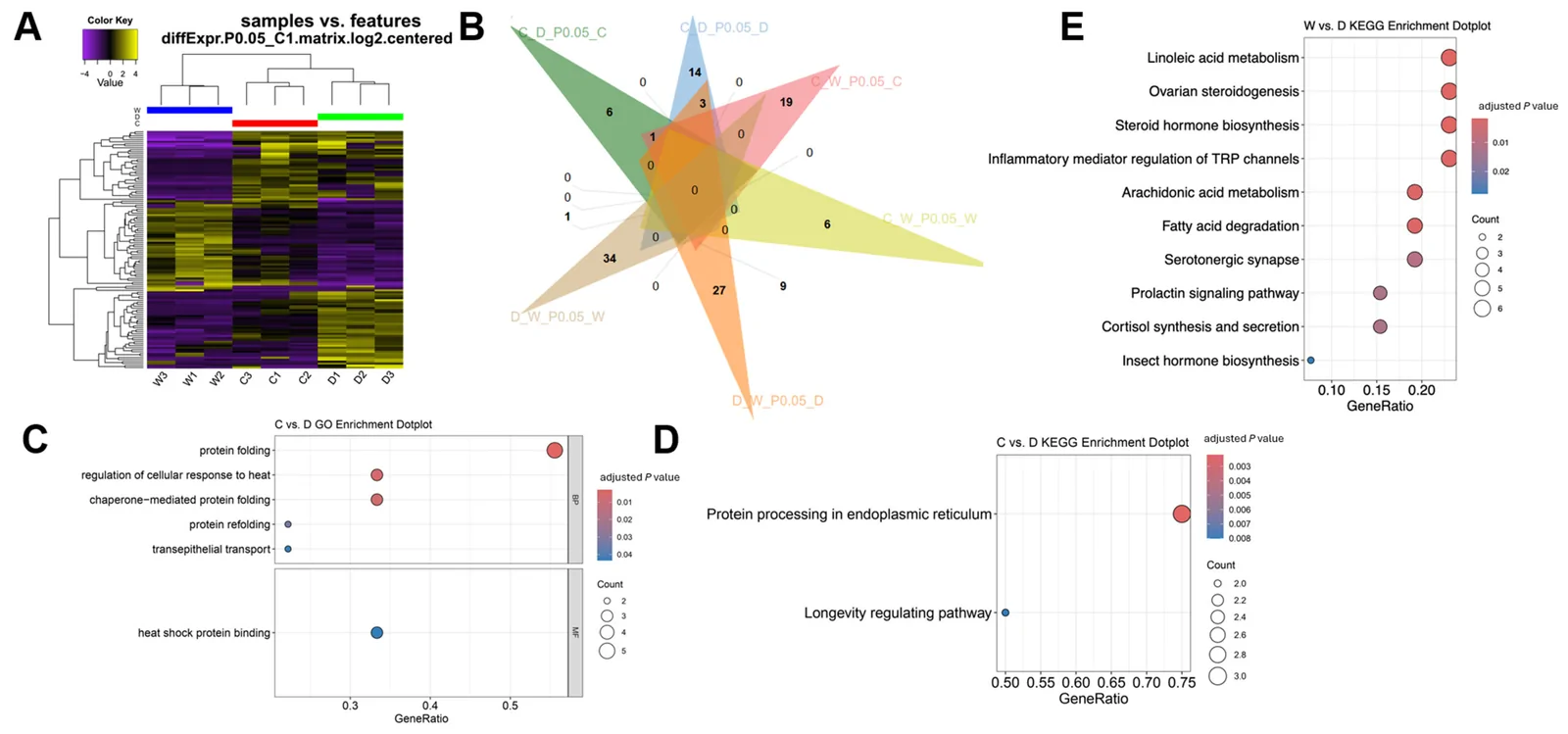
Transcriptomic profiling of earthworms under different moisture conditions. (**A**) Hierarchical clustering heatmap of differentially expressed genes (DEGs) revealed from the three moisture treatments. (**B**) Venn diagram illustrating the common and unique DEGs at different treatments. (**C**,**D**) Gene Ontology (GO) (**C**) and KEGG pathway enrichment (**D**) analyses of DEGs between 80% WHC and 30% WHC. (**E**) KEGG pathway enrichment analysis of DEGs between 100% WHC and 30% WHC.

A total of 72,716,287 transcriptome sequencing reads were generated in this study (Supplementary Table S4). After quality control screening, a total of 68,974,933 clean reads with Q30 scores above 97.39% for all samples were further used for transcriptomic analyses (Supplementary Table S5). The constructed transcriptomes and the predicted proteomes are in good completeness as indicated by BUSCO completeness assessments (97.8% and 98.1%, respectively, Supplementary Table S6). Gene Ontology (GO) classification assigned ~32.9% of transcripts to at least one GO category (Supplementary Table S7), while KEGG pathway analysis mapped transcripts to various metabolic and signalling pathways (Supplementary Figure S2; Supplementary Table S8). Clustering and principal component analysis (PCA) showed clear grouping of samples in different moisture treatments (Figure 1A; Supplementary Figures S3 and S4). Hereafter, C, D, and W denote control (80% WHC), dry (30% WHC), and water-saturated (100% WHC) groups, respectively. Among the 120 unique differentially expressed genes (DEGs) identified across all pairwise comparisons, no single DEG was shared among all three comparisons. However, fourteen DEGs were shared between specific pairwise comparisons(Figure 1B; Supplementary Table S9).

Comparison of the samples in the control (80% soil water-holding capacity (WHC), C group) with those in the dry condition (30% WHC, D group) revealed that a total of 17 and seven transcripts were up- and downregulated in the D group, respectively. These include the upregulation of heat shock proteins sHsp20 and Hsp70 (Supplementary Figures S5 and S6). Functional enrichment analyses further identified that biological processes involved in protein folding and endoplasmic reticulum protein processing were significantly represented (Figure 1C,D; Supplementary Tables S10 and S11; Supplementary Figures S7 and S8). On the other hand, comparison of samples in the wet (100% soil water-holding capacity (WHC), W group) with those in the dry condition (30% WHC, D group) revealed a total of 35 and 39 transcripts were up- and downregulated in the W group, respectively. KEGG pathway enrichment analyses highlighted that genes involved in lipid metabolism, steroid hormone biosynthesis, and amino acid transport were involved (Figure 1E; Supplementary Tables S12 and S13; Supplementary Figures S9 and S10). Further comparison of samples between 80% WHC and 100% WHC identified a total of 36 DEGs (29 upregulated and 7 downregulated in the 80% WHC group), and no significantly enriched gene pathway was identified (Supplementary Figure S11; Supplementary Table S14). 

**Figure 2:**
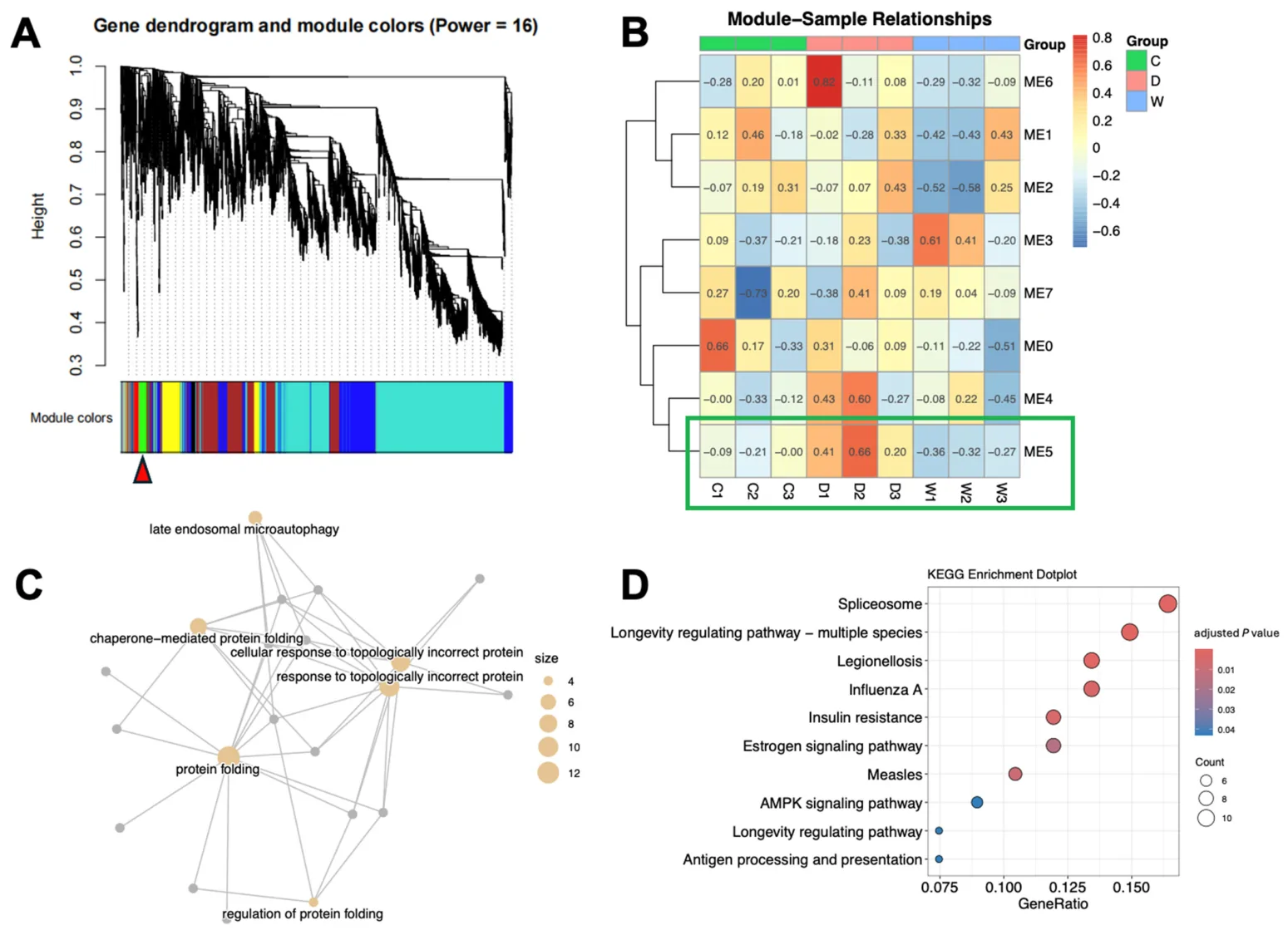
Weighted gene co-expression network analysis (WGCNA) of core desiccation-responsive module. (**A**) Hierarchical clustering dendrogram of the top 5000 most variable genes (soft-thresholding power = 16). The arrowhead shows the position of ME5 module. (**B**) Module–sample relationship heatmap showing ME5 module (box) exhibits the most significant positive correlation among different moisture groups. (**C**) Network visualization of enriched GO terms within the ME5 module. (**D**) KEGG pathway enrichment analysis of ME5 module.

Weighted gene co-expression network analysis revealed eight distinct modules (Supplementary Figure S12), and the ME5 module (containing 123 genes) showed the strongest positive correlation with the moisture conditions (*p* = 0.0022; Figure 2A,B; Supplementary Table S15). ME5 showed gene pathway enrichment in maintaining cellular proteostasis under environmental stress, and based on intramodular connectivity and gene significance, *Hsp70s* (TRINITY_DN3554_c0_g3, TRINITY_DN432_c0_g2, TRINITY_DN4278_c0_g1, TRINITY_DN2843_c0_g1) were identified as the core set of highly connected hub genes in this module. GO analysis on different modules showed that genes involved in protein folding, chaperone-mediated responses, and the clearance of topologically incorrect proteins via late endosomal microautophagy are involved (Figure 2C; Supplementary Table S16; Supplementary Figure S13), which is similar to the KEGG pathway analysis highlighting significant enrichment in the longevity regulating pathway and energy-sensing networks (e.g., AMPK and insulin resistance pathways) (Figure 2D; Supplementary Table S17). 

**Figure 3:**
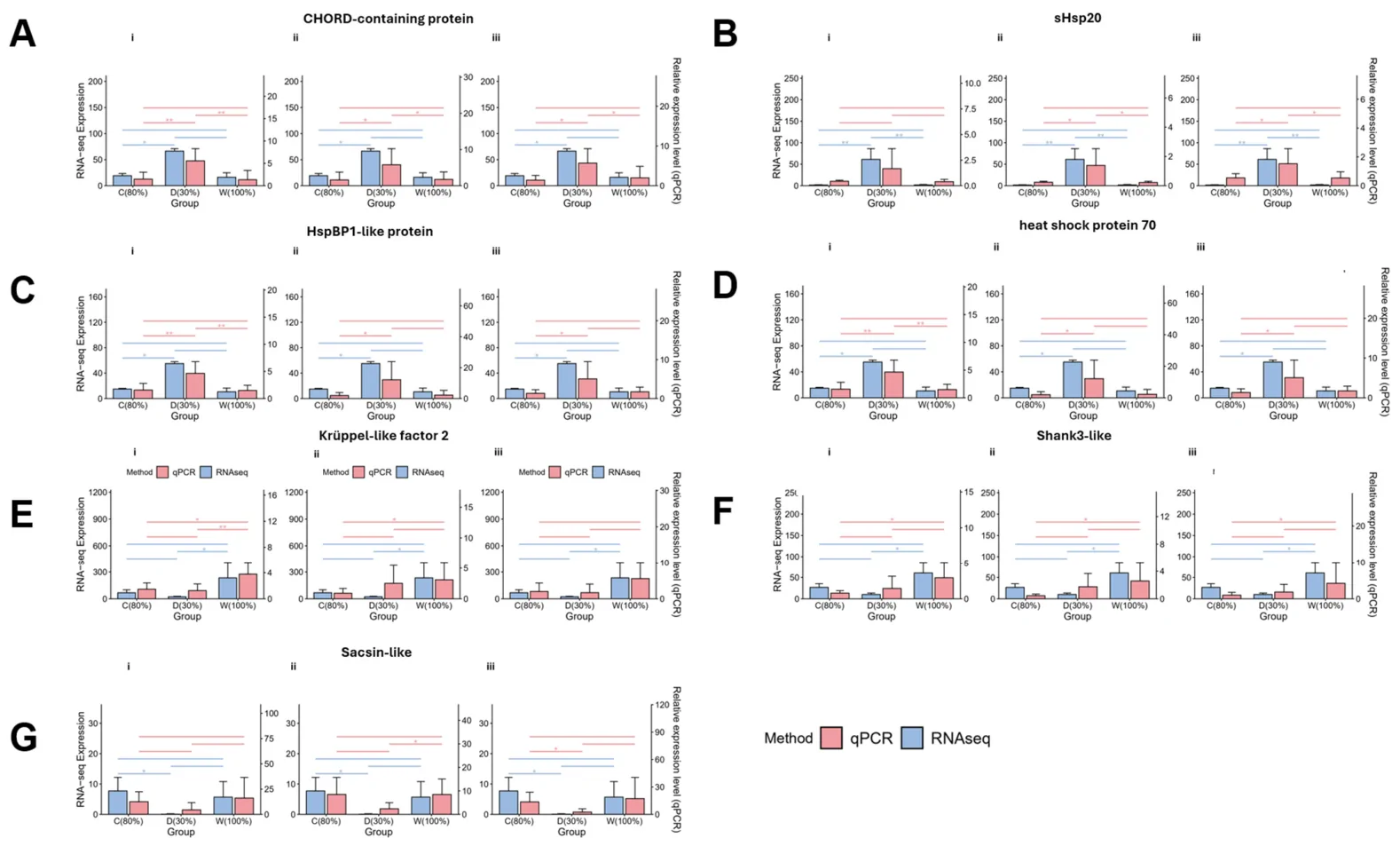
Quantitative real-time PCR validation of DEGs identified in transcriptomic analyses. Relative expression levels of differentially expressed genes at different moisture treatments: dry 30% WHC (D group), control 80% WHC (C group), and saturation 100% WHC (W group). (**A**–**D**) Genes upregulated at D group: CHORD-containing protein (**A**), sHsp20 (**B**), HspBP1-like protein (**C**), and Hsp70 (**D**). (**E**,**F**) Genes upregulated at W group: Krüppel-like factor (**E**) and Shank3-like (**F**). (**G**) Gene downregulated in the D group: Sacsin-like. Within each gene panel, i, ii, and iii denote qRT-PCR normalization to housekeeping gene: GAPDH, ribosomal protein L11, and ribosomal protein L3, respectively. Data are presented as mean ± S.D. (*n* = 8 biological replicates each with 2 technical replicates). Red and blue lines indicate pairwise comparison in qRT-PCR and transciptomic analyses, respectively. Statistical significance was determined using Welch’s *t*-test: * *p* < 0.05, ** *p* < 0.01.

To validate the key DEGs identified above, we performed qRT-PCR, and seven of the eight chosen tested genes showed consistent expression trends with the RNA-seq data analyses (Figure 3; Supplementary Figure S14). These DEGs included cysteine- and histidine-rich domain (CHORD)-containing protein (TRINITY_DN989_c7_g1), sHsp20 (TRINITY_DN752_c0_g1), HspBP1-like protein (TRINITY_DN4892_c0_g1), and Hsp70 (TRINITY_DN3554_c0_g3), all of which were upregulated under dry 30% WHC (D group) (Figure 3A–D); Krüppel-like factor 2 (KLF2) (TRINITY_DN1458_c0_g1) and Shank3-like (TRINITY_DN7916_c0_g1), which were upregulated under 100% WHC (W group) (Figure 3E,F); and Sacsin-like (TRINITY_DN44067_c0_g1), which was downregulated under 30% WHC (D group) (Figure 3G). Minor discrepancies in specific pairwise significance between the two platforms are expected given inherent differences in sensitivity, dynamic range, and normalization. Overall, the strong concordance in expression trends confirms the reliability of the transcriptomic data.

### 3.2. Differentially Expressed microRNAs in Anterior Tissues at Different Soil Moistures

**Figure 4:**
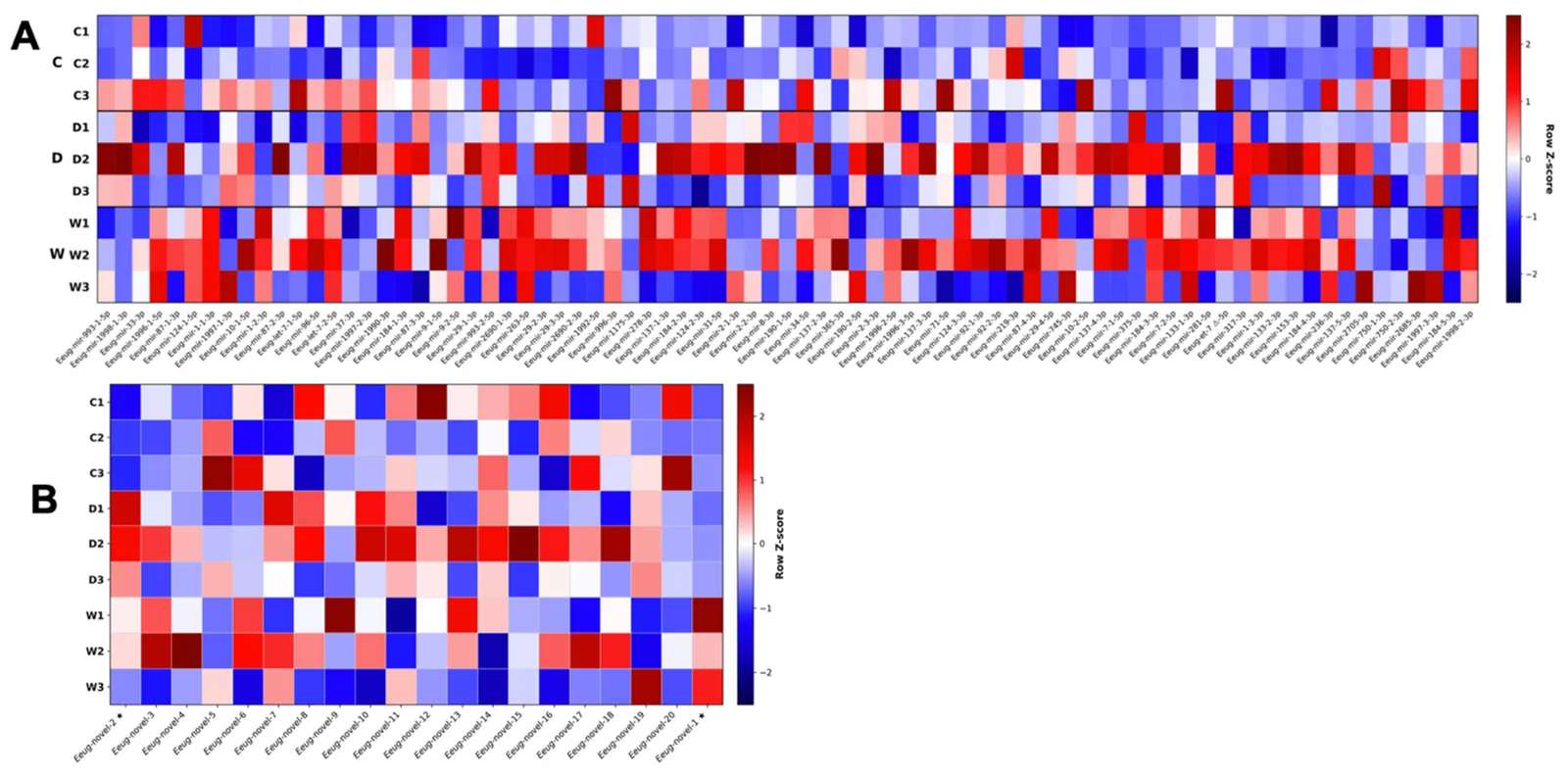
Expression profiles of known and novel microRNAs across moisture treatments. (**A**) Expression heatmap of the 79 known microRNAs (mature arm). (**B**) Expression heatmap of the 20 novel microRNAs (mature arm); Eeug-novel-1 and Eeug-novel-2 are marked with asterisks.

A total of 141,056,726 raw small RNA sequencing reads were generated in this study (Supplementary Table S18), and mapping the cleaned reads to the published draft genome allowed us to reveal a total of 79 known and 20 lineage-specific microRNAs (Figure 4A,B; Supplementary Tables S19 and S20). By comparing their expression levels across different moisture treatments, we identified two differentially expressed microRNAs, both of which were lineage-specific microRNAs: Eeug-novel-1 and Eeug-novel-2. To understand the potential microRNA:mRNA gene regulatory networks of these two DE-microRNAs, we performed in silico target prediction and identified 1389 potential interacting gene partners in the transcriptomes (Supplementary Figure S15). Among them, six of these genes were differentially expressed at different moisture treatments (Supplementary Table S21).

**Figure 5:**
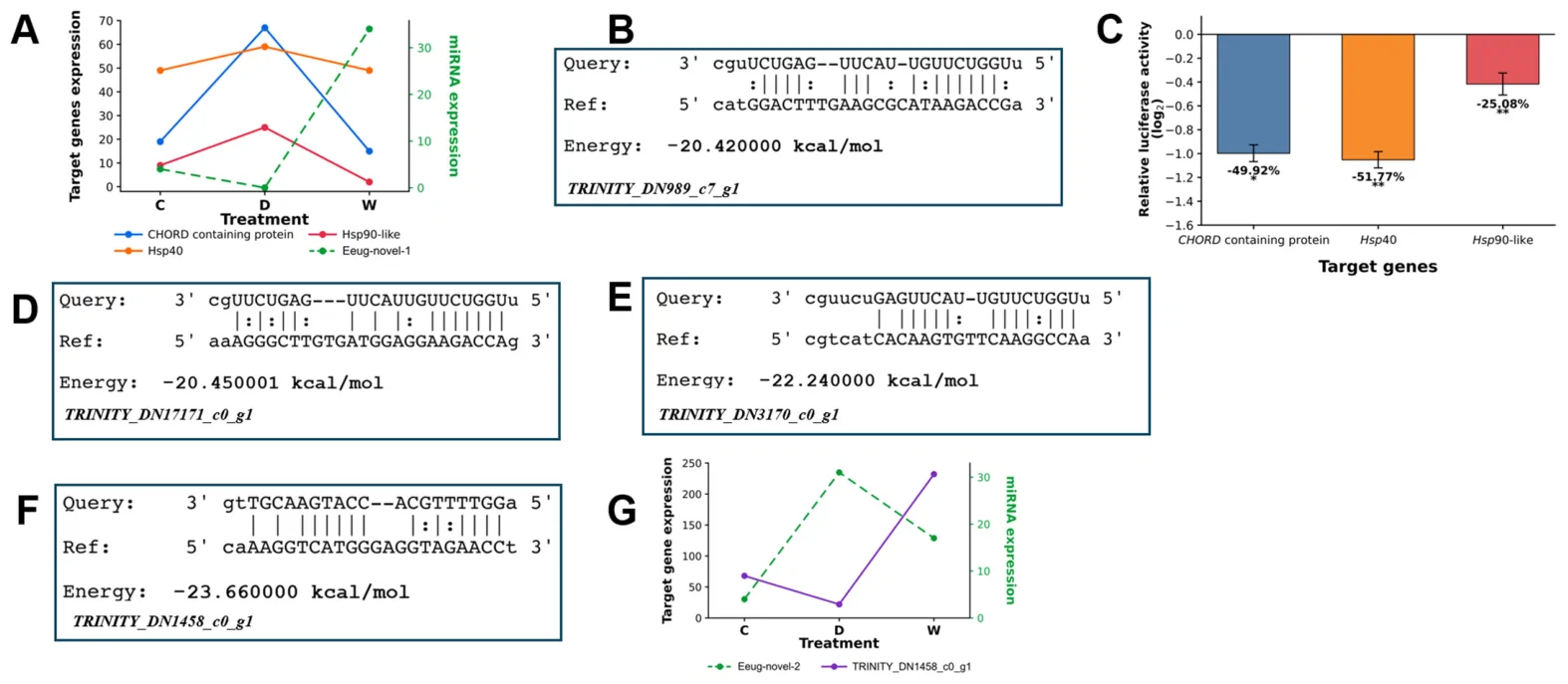
Predicted and experimentally supported interactions of the two moisture-responsive novel microRNAs. (**A**) Expression profiles of Eeug-novel-1 (dashed green line) and its three candidate chaperone targets across C, D, and W. (**B**) Predicted binding between Eeug-novel-1 and the CHORD-containing protein transcript (TRINITY_DN989_c7_g1). (**C**) Dual-luciferase reporter assay for the CHORD-containing protein, Hsp40, and Hsp90-like target regions. (**D**,**E**) Predicted binding of Eeug-novel-1 to Hsp90-like (**D**) and Hsp40 (**E**). (**F**) Predicted binding between Eeug-novel-2 and KLF2 (TRINITY_DN1458_c0_g1). (**G**) Expression profiles of Eeug-novel-2 (dashed green line) and KLF2 across C, D, and W. Relative luciferase activity is shown as the Renilla/firefly ratio normalized to the control; statistical significance was determined using Welch’s *t*-test (* *p* < 0.05, ** *p* < 0.01).

Gene pathway analyses of the Eeug-novel-1 predicted targets revealed enrichment of pathways related to nutrient/energy sensing (insulin signalling, mTOR, AMPK, and FoxO signalling pathways) and cytoskeletal regulation (Supplementary Tables S22 and S23). Notably, Eeug-novel-1 and its predicted target CHORD-containing protein (TRINITY_DN989_c7_g1) exhibited opposite expression trends across moisture treatments (Figure 5A). Their interaction was supported by target-site prediction (Figure 5B), qRT-PCR (Figure 3A), LAMP (Supplementary Figure S16), and the dual-luciferase assay (Figure 5C). Interestingly, two additional molecular chaperones residing in the same ER protein processing pathway—Hsp90-like (TRINITY_DN17171_c0_g1) and Hsp40 (TRINITY_DN3170_c0_g1), were also found to be targets of Eeug-novel-1 as indicated by the prediction, pull-down and luciferase assays (Figure 5C–E; Supplementary Figures S17 and S18).

For the other differentially expressed microRNA, Eeug-novel-2, this microRNA was significantly upregulated in comparison with dry 30% WHC (D group) to control 80% WHC (C group). Gene pathway analyses of its predicted target genes identified no significant enrichment. Focusing on the interaction between this microRNA and the differentially expressed genes under different moisture treatments, two potential targets were identified, including KLF2 (TRINITY_DN1458_c0_g1) (Figure 5F), which was significantly upregulated under saturation 100% WHC (W group) relative to dry 30% WHC (D group) and independently validated using qRT-PCR (Figure 3E), and a hypothetical protein (TRINITY_DN11015_c2_g1) of unknown function. Consistent with canonical microRNA-mediated repression, Eeug-novel-2 and KLF2 exhibited inverse expression trends across the moisture gradient (Figure 5G). LAMP confirmed that Eeug-novel-2 could interact with KLF2 (Supplementary Figure S19). Notably, KLF2 shares the Gene Ontology term “negative regulation of supramolecular fiber organization” with heat shock protein family members identified above. Whether and how these two microRNAs cooperate under moisture stress in *E. eugeniae* remains to be further investigated.

## 4. Discussion

This study utilized the tropical earthworm *E. eugeniae* to explore the effects of soil moisture on the anterior tissues, which provides new insights into the adaptation of the microRNA:mRNA network in earthworms. Prior to this study, there were limited studies investigating the mRNA transcriptomic response in earthworms facing desiccation. In *Eisenia fetida*, an earthworm species originally native to Europe, heat shock proteins and hypoxia-related genes were found to be involved in desiccation [7]. Our data are consistent with the previous study and show that the expression levels of several heat shock protein genes (sHsp20, HspBP1-like protein, Hsp70) differed significantly in the tropical species *E. eugeniae* across different soil moisture conditions. 

Small heat shock proteins such as sHsp20 are known to capture misfolding intermediates through ATP-independent holdase activity in the chaperone cascade [45], and these substrates will be transferred to the Hsp70 machinery, while Hsp40 delivers clients and stimulates ATPase activity [46]. These data suggest that certain genetic responses to soil moisture across earthworm species could be a conserved mechanism. 

**Figure 6:**
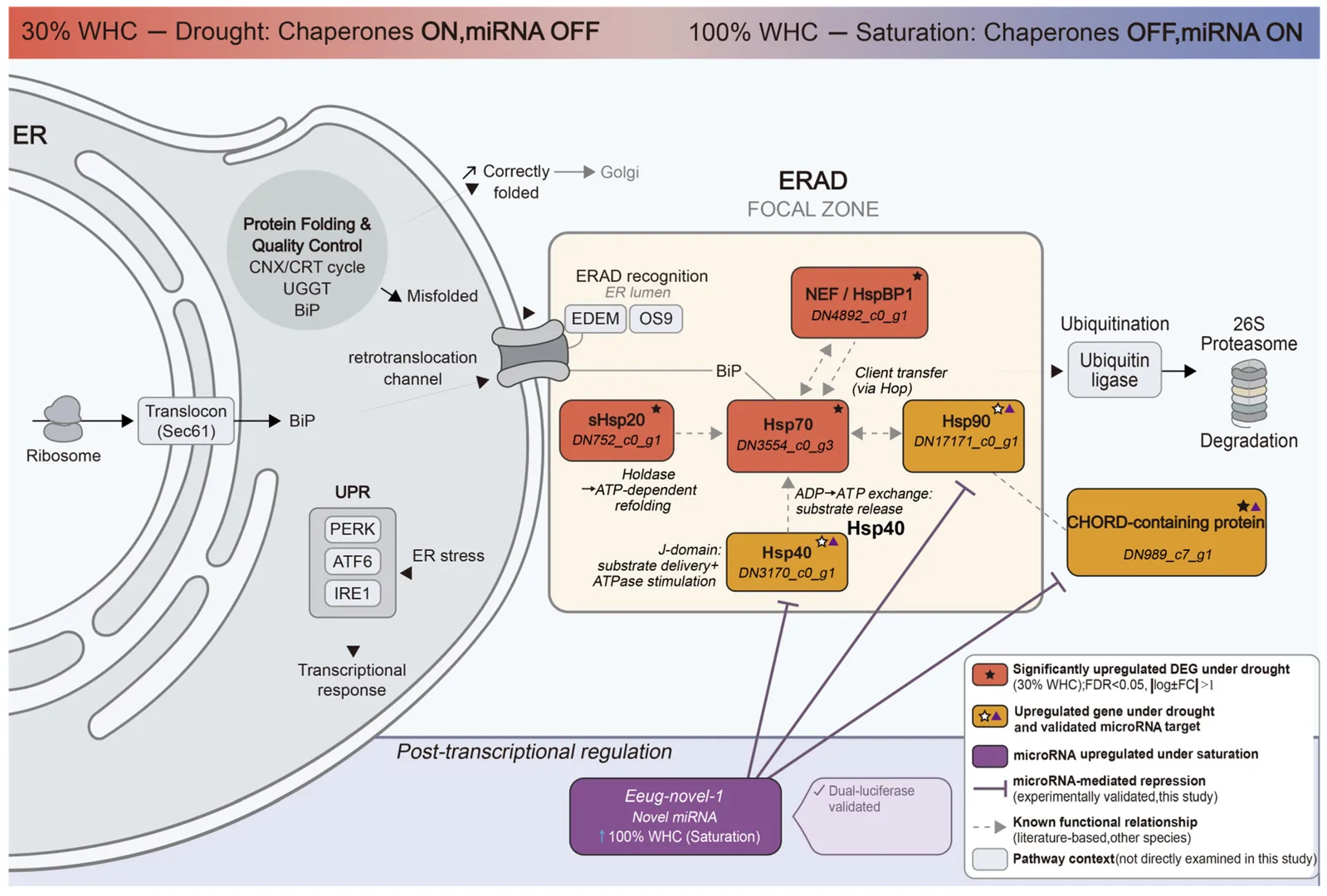
Schematic diagram showing the proposed model of post-transcriptional regulation in *Eudrilus eugeniae* at different soil moisture conditions. The blue upward arrow denotes upregulation of Eeug-novel-1 expression under 100% WHC.

In addition, our experiments covering multiple moisture conditions revealed that *E. eugeniae* responds to desiccation via a focused network on proteostasis involving CHORD-containing proteins. Completion of the Hsp70 refolding cycle generally requires nucleotide exchange factors such as HspBP1 [47], and Hsp90 and its co-chaperones triage terminally misfolded substrates toward degradation when refolding is unsuccessful [48]. The CHORD-containing protein interacts with the N-terminal ATPase domain of Hsp90 and functions as established Hsp90 co-chaperones [49,50]. Simultaneous upregulation of components spanning this entire relay from the initial holdase (sHsp20), through the central ATP-dependent refolding machinery (Hsp40, Hsp70, HspBP1), to the downstream triage system (Hsp90 and CHORD-containing co-chaperone), collectively suggests that desiccation co-activates the complete chaperone cascade in response to moisture changes in earthworms (Figure 6). Since this interpretation is based on co-expression patterns and functional relationships established in other organisms, further validation of these chaperones in *E. eugeniae* will be needed to understand their roles in the presence of different soil moisture/desiccation.

Another key finding of this study is the understanding of how microRNAs regulate desiccation responses in earthworms. MicroRNAs in earthworms remain relatively understudied, with previous work primarily focused on profiling microRNA expression during tissue regeneration [13]. Similarly, a recent genome resource for the annelid *Platynereis* catalogued 105 microRNA loci but did not examine their expression under abiotic stress [51]. This study reveals the first set of differentially expressed microRNAs involved in regulation of the chaperone components (CHORD-containing protein, Hsp40, and Hsp90-like) at different soil moistures in earthworms. Notably, the chaperone targets regulated by Eeug-novel-1 (Hsp40 and Hsp90-like) are different candidates in the genetic pathway compared to the differentially expressed heat shock protein genes identified by mRNA transcriptomics (sHsp20, Hsp70, HspBP1-like). This suggests holistic cooperation between transcriptional and post-transcriptional mechanisms in earthworms facing soil moisture variation. Given that the differentially expressed microRNAs identified here are lineage-specific, whether the microRNA:mRNA regulatory network observed represents a condition- or species-specific scenario, or is more generally applicable to different earthworm species, remains to be tested.

From ecological and environmental perspectives, earthworm ecosystem services—including soil structure maintenance, nutrient cycling, and contributions to crop productivity—are directly constrained by soil moisture [3,5]. Thus, characterizing the responses of earthworms at different moisture conditions will be relevant for understanding their population dynamics in the field. This study advances our current understanding of the mRNA and microRNA regulatory networks under moisture stress in a tropical earthworm species. Further studies on the combined effects of temperature and water availability on different species of earthworms will be needed to fully understand the effect of climate change on their ecosystem services at different habitats in different geographical regions. 

## 5. Conclusions

This study provides the first evidence for the involvement of microRNAs and molecular chaperone genes in the responses of earthworms to different soil moisture conditions.

## Data Availability

The raw RNA-seq and small RNA-seq data generated in this study have been deposited in CUHK Research Data Repository database and are publicly available at https://doi.org/10.48668/Q03ZPY (accessed on 6 August 2026).

## Supplementary Materials

The following supporting information can be downloaded at: https://www.mdpi.com/article/10.3390/biology15161387/s1.

## Abbreviations

3′UTR	3′ untranslated region
AMPK	AMP-activated protein kinase
ANOVA	analysis of variance
bp	base pair(s)
BUSCO	Benchmarking Universal Single-Copy Orthologs
C	control condition at 80% WHC
cDNA	complementary DNA
CHORD	cysteine- and histidine-rich domain
CHORDC1	CHORD-containing protein 1
COI	cytochrome c oxidase subunit I
D	dry condition at 30% WHC
CPM	counts per million
DEGs	differentially expressed genes
ER	endoplasmic reticulum
FDR	false discovery rate
FoxO	forkhead box O
GFF	General Feature Format
GO	Gene Ontology
Hsp/HSP	heat shock protein
KEGG	Kyoto Encyclopedia of Genes and Genomes
KLF	Krüppel-like factor
LAMP	labelled microRNA pull-down assay
MAD	median absolute deviation
MFE	minimum free energy
mRNA	messenger RNA
nt	nucleotide(s)
ORF	open reading frame
PCA	principal component analysis
PCR	polymerase chain reaction
qRT-PCR	quantitative real-time polymerase chain reaction
RNA	ribonucleic acid
RNA-seq	RNA sequencing
RSEM	RNA-Seq by Expectation-Maximization
sHsp	small heat shock protein
sRNA	small RNA
TMM	Trimmed Mean of M-values
WGCNA	weighted gene co-expression network analysis
W	water-saturated condition at 100% WHC
WHC	water-holding capacity
