# Supplementary material for: Integrated Analyses of mRNA and microRNA Regulatory Networks at Different Soil Moisture in Tropical Earthworm Eudrilus eugeniae
Source: Biology (Basel). 2026 Aug 13;15(16):1387. doi: 10.3390/biology15161387 (PMC13509270; doi:10.3390/biology15161387)
Supplement: Supplementary file 1 [file biology-15-01387-s001.zip › Supplementary Figure.pdf]

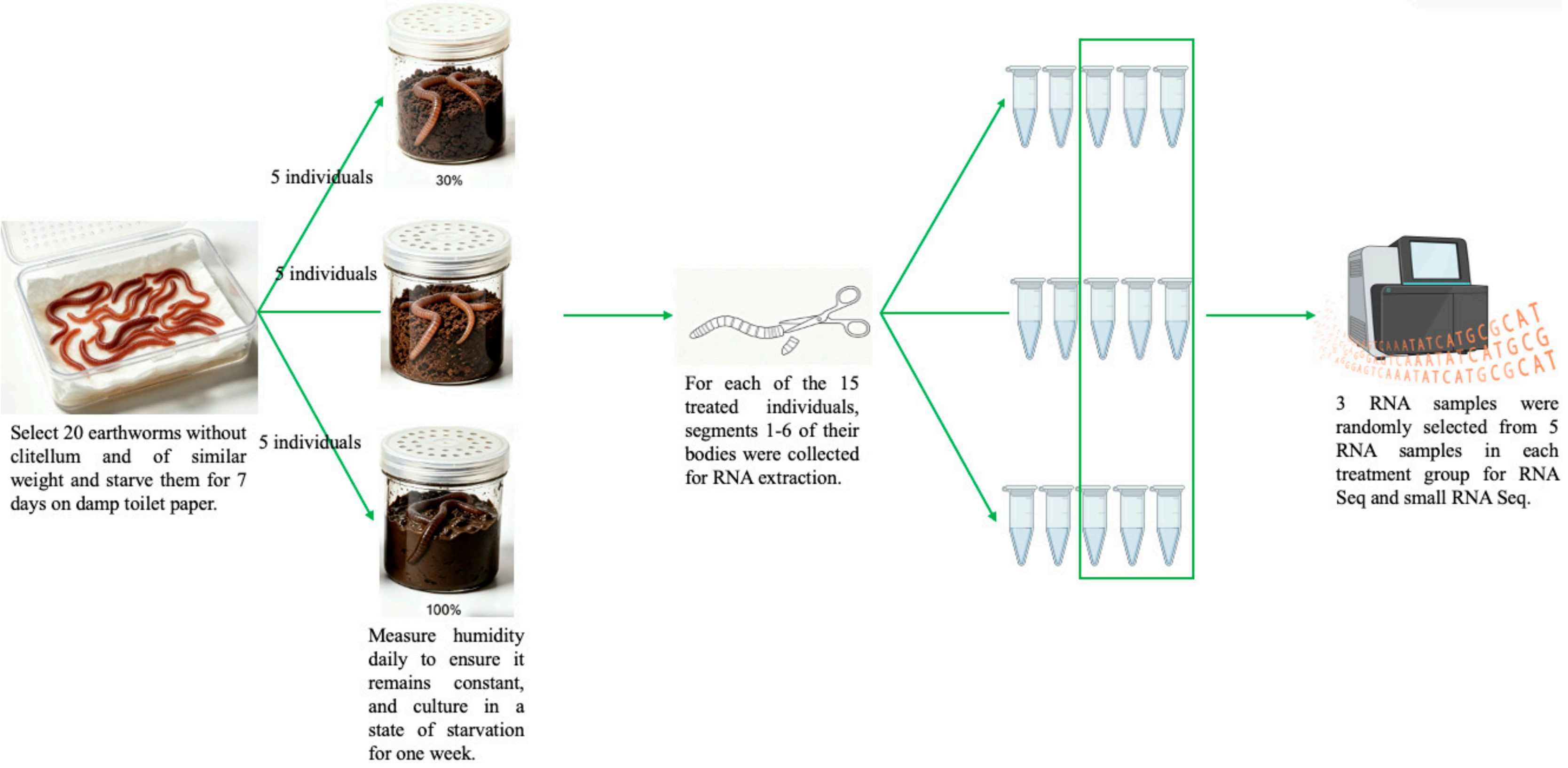

Supplementary Figure S1. Experimental design and soil moisture treatments. *E. eugeniae* individuals (n = 5 per group) were exposed to three soil water-holding capacity (WHC) regimes for one week: dry 30% WHC (D group), control 80% WHC (C group), and water-saturated 100% WHC (W group). Anterior segments (segments 1–6) were dissected for paired RNA and small RNA sequencing. Preliminary trials indicated a critical survival threshold, with 100% mortality occurring within 3 days at 25% WHC.

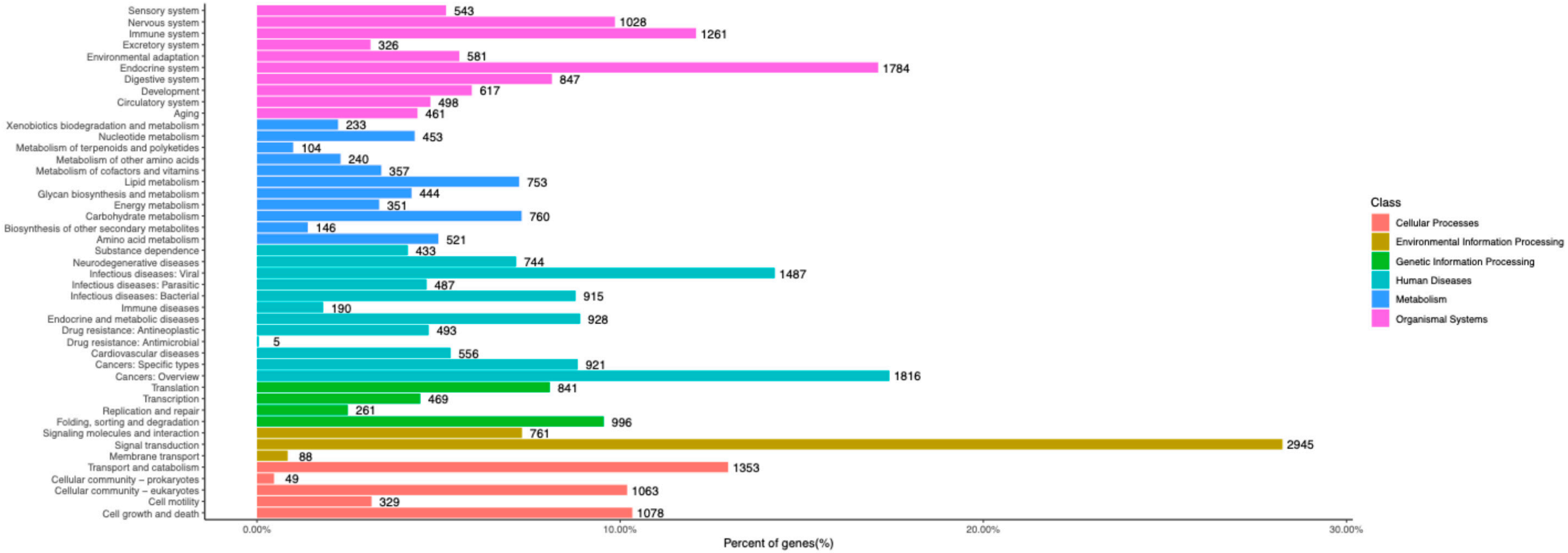

Supplementary Figure S2. Functional classification of the assembled *E. eugeniae* transcriptome by KEGG pathway annotation. Transcripts with significant hits from eggNOG-mapper were assigned to KEGG pathway categories spanning metabolism, environmental information processing, cellular processes, and organismal systems. Bar lengths indicate the number of transcripts mapped to each category.

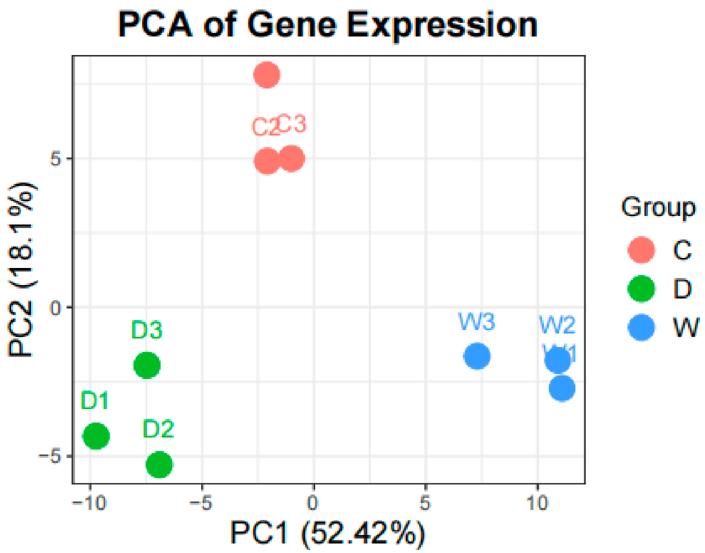

Supplementary Figure S3. Principal component analysis (PCA) of differentially expressed genes (DEGs) demonstrating distinct clustering of samples driven by moisture levels.

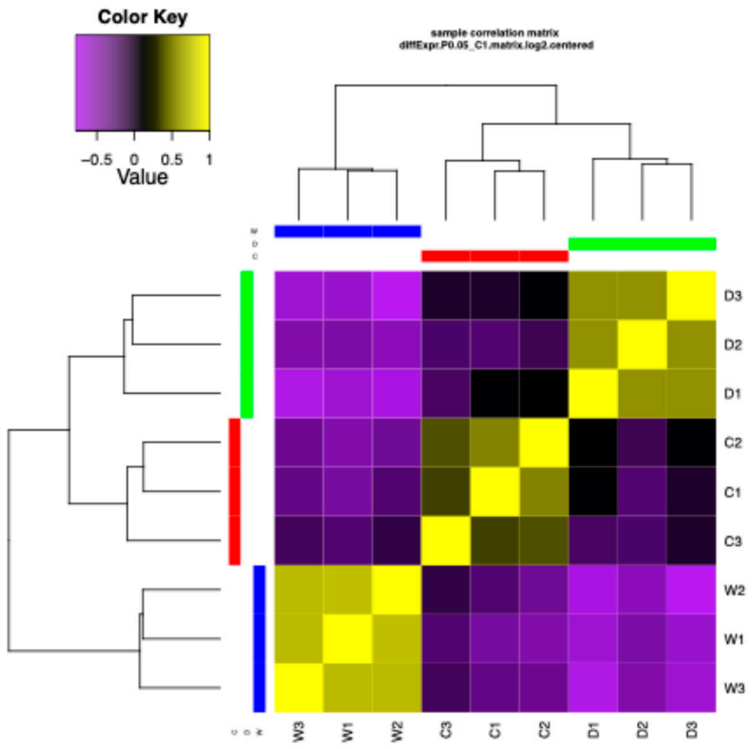

Supplementary Figure S4. Matrix of global transcriptome expression profiles across all nine sequenced samples. The clear separation of samples confirms that moisture regime is the dominant source of transcriptomic variation.

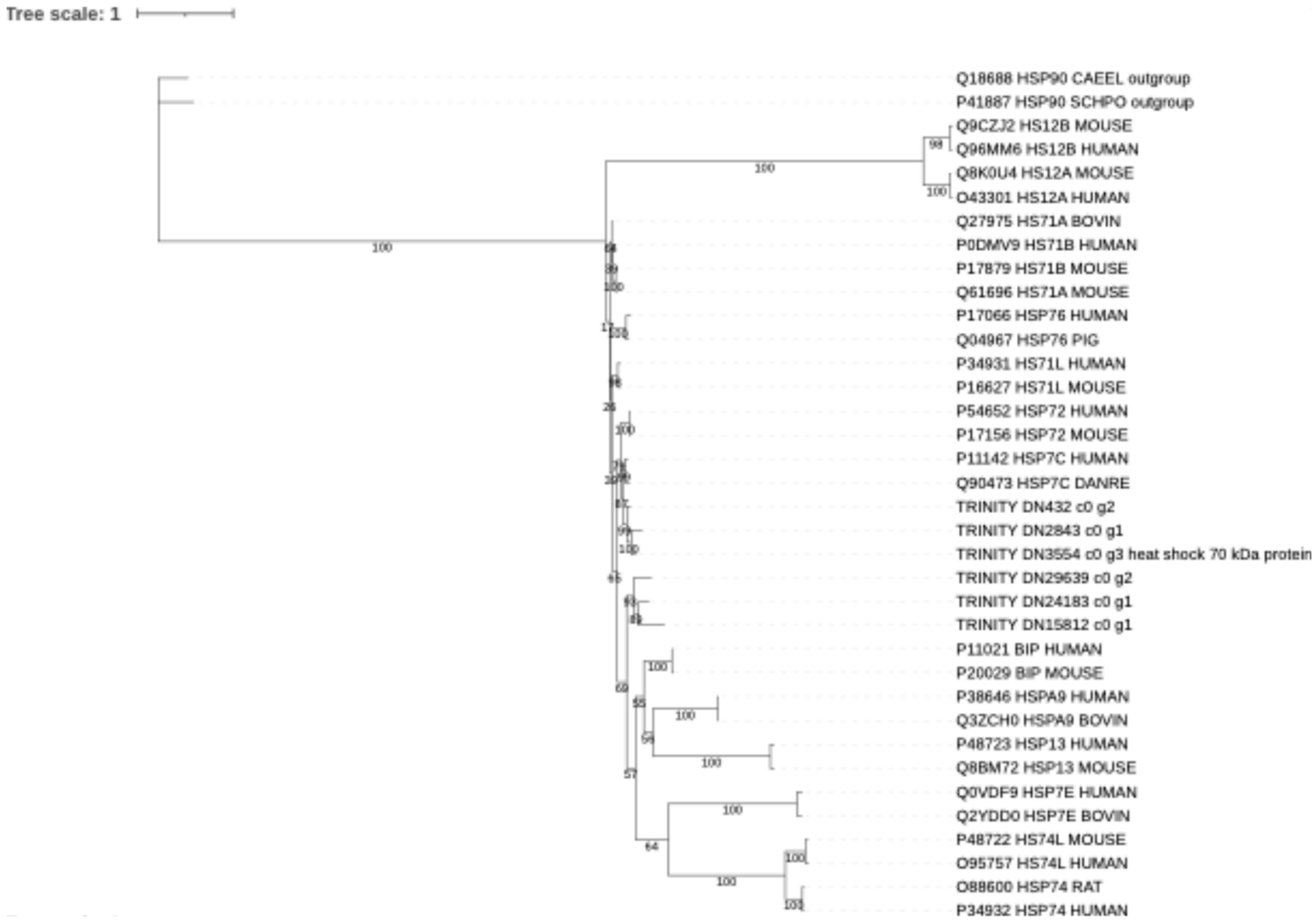

Supplementary Figure S5. Maximum-likelihood tree of *Hsp70* family members in *E. eugeniae* inferred from IQ-TREE (100 bootstrap values).

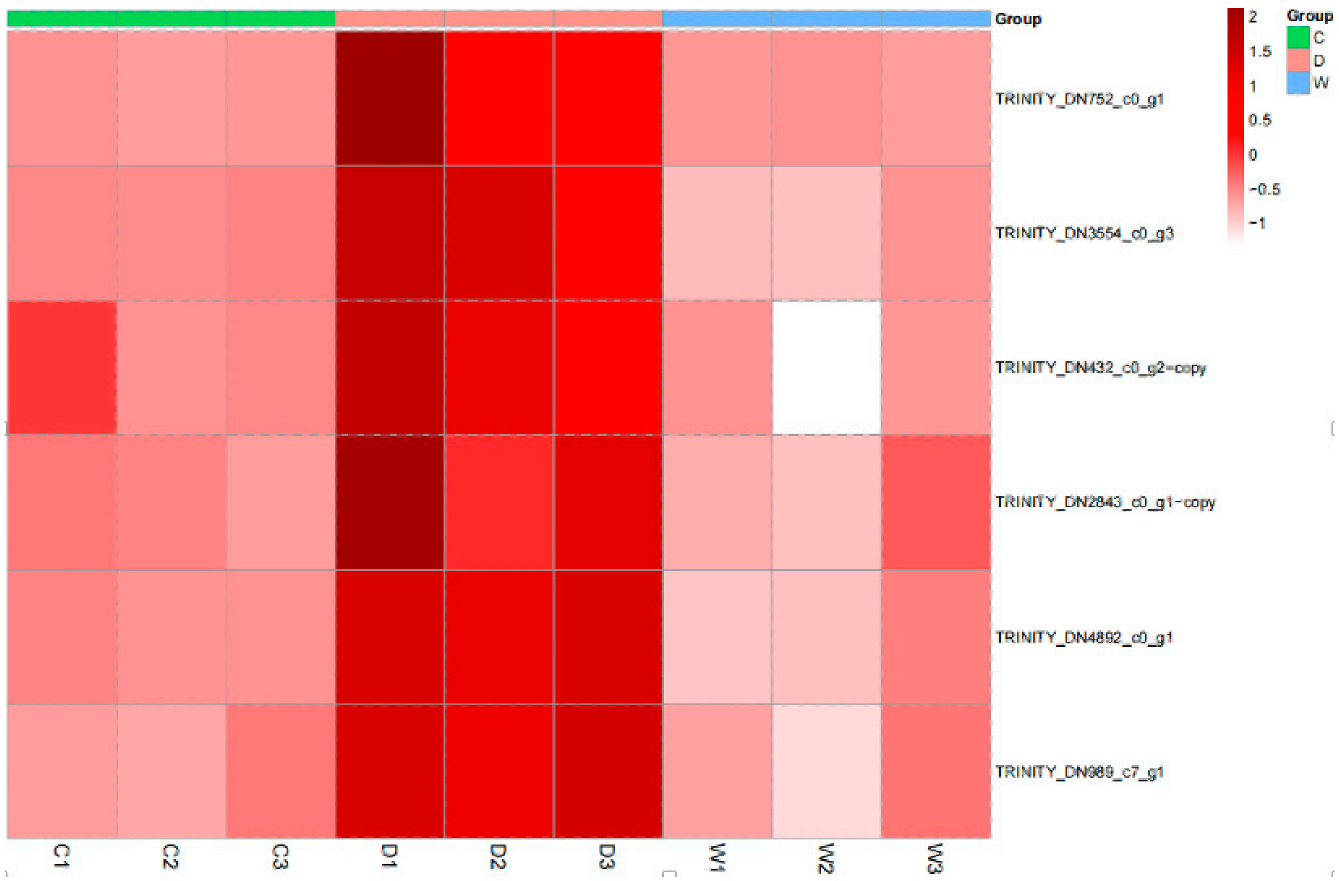

Supplementary Figure S6. Heatmap showing the expression profiles of heat shock protein genes across the treatments.

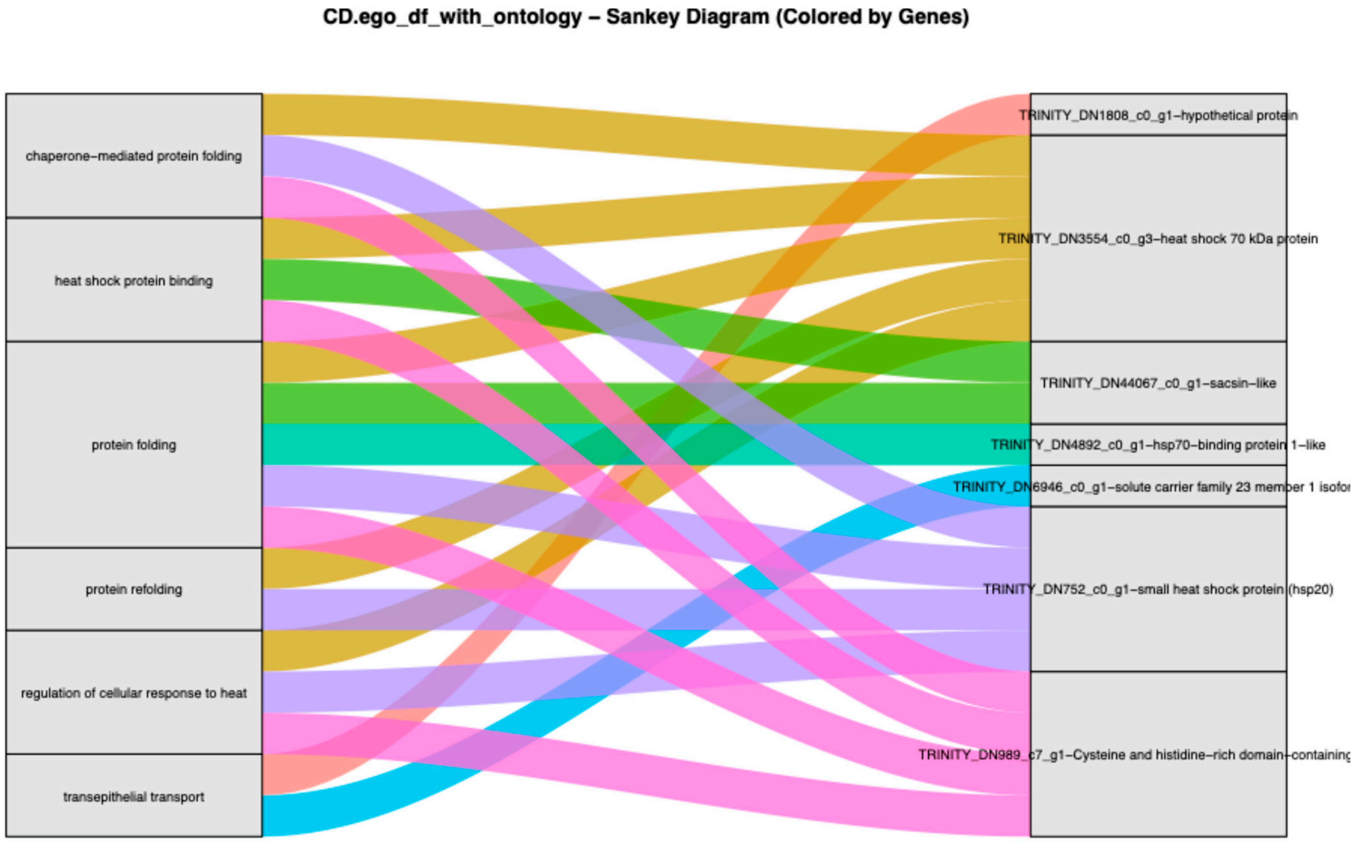

Supplementary Figure S7. Sankey (alluvial) diagram illustrating the gene-to-term mapping for significantly enriched Gene Ontology (GO) categories among differentially expressed transcripts between the control 80% WHC (C) and dry 30% WHC (D) groups. Right nodes represent individual genes and left nodes represent enriched GO terms; connecting ribbons indicate membership of each gene within the corresponding GO category, revealing the extent of gene sharing across functional terms.

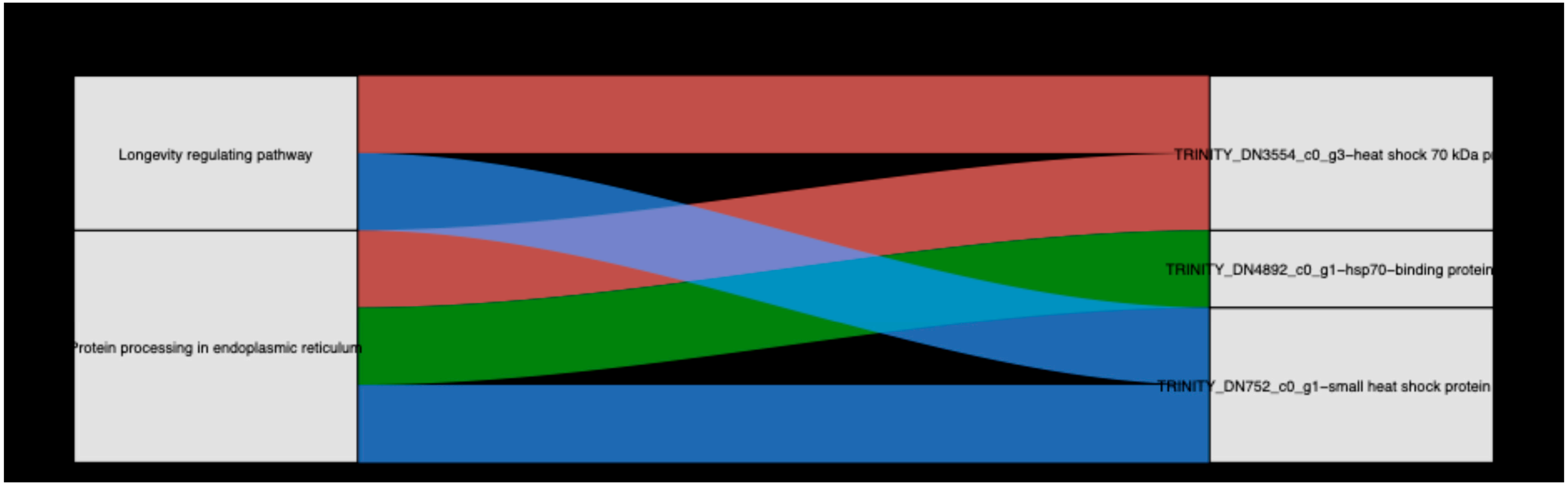

Supplementary Figure S8. Sankey (alluvial) diagram illustrating the gene-to-pathway mapping for significantly enriched KEGG pathways among differentially expressed transcripts between the control 80% WHC (C) and dry 30% WHC (D) groups. Right nodes represent individual genes and left nodes represent enriched KEGG pathways; connecting ribbons show which genes contribute to each pathway, highlighting the distribution of differentially expressed genes across functional modules.

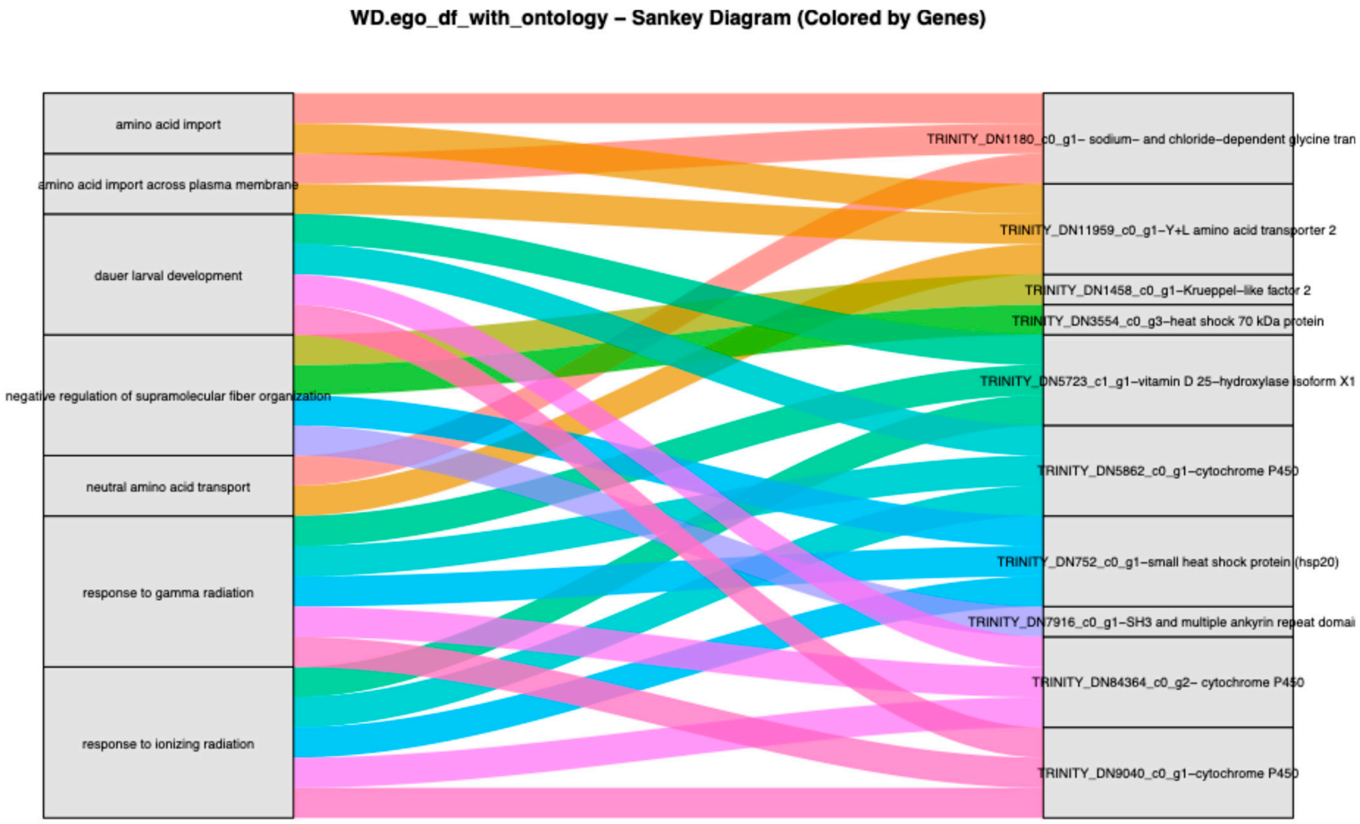

Supplementary Figure S9. Sankey (alluvial) diagram illustrating the gene-to-term mapping for significantly enriched Gene Ontology (GO) categories among differentially expressed transcripts between the water-saturated 100% WHC (W) and dry 30% WHC (D) groups. Right nodes represent individual genes and left nodes represent enriched GO terms; connecting ribbons indicate the contribution of each gene to the corresponding GO category.

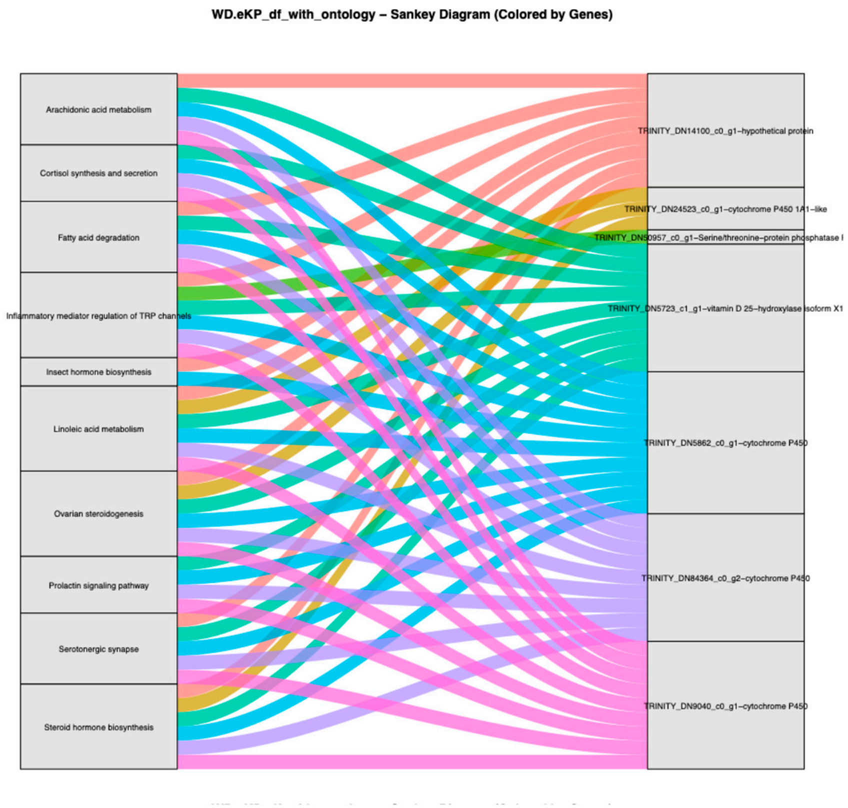

Supplementary Figure S10. Sankey (alluvial) diagram illustrating the gene-to-pathway mapping for significantly enriched KEGG pathways among differentially expressed transcripts between the water-saturated 100% WHC (W) and dry 30% WHC (D) groups. Right nodes represent individual genes and left nodes represent enriched KEGG pathways; connecting ribbons indicate the contribution of each gene to each pathway, underscoring the prominence of lipid metabolism, steroid biosynthesis, and cytochrome P450 activity in this comparison.

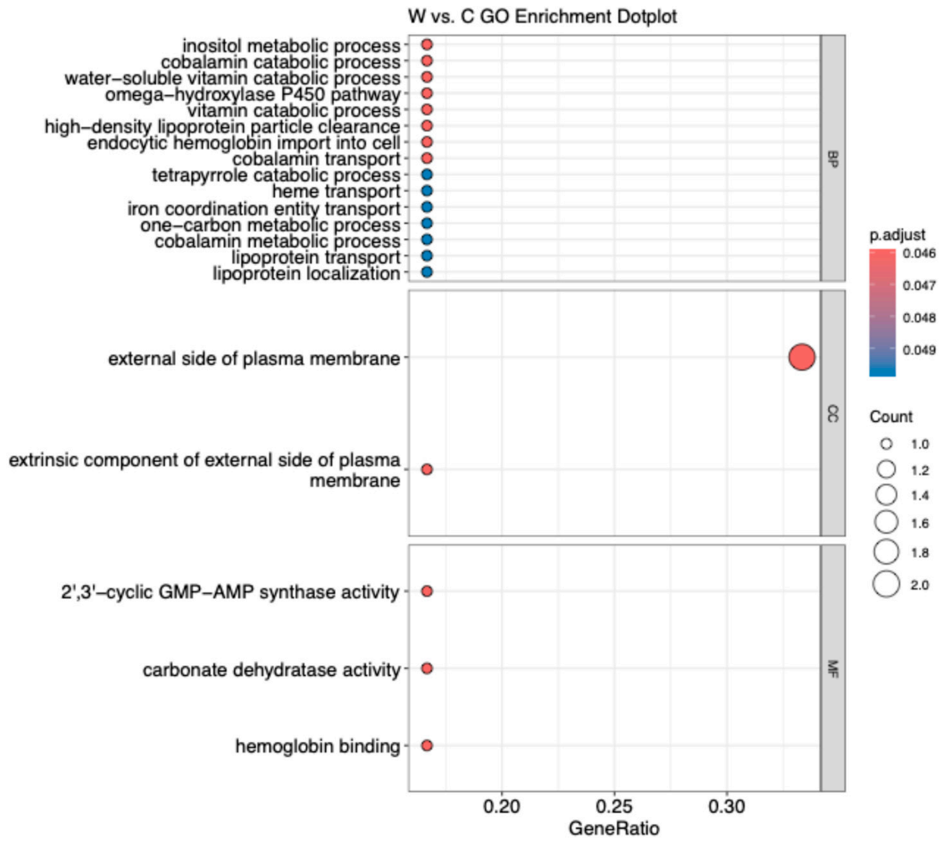

Supplementary Figure S11. Functional enrichment analysis of differentially expressed transcripts between the control 80% WHC (C) and water-saturated 100% WHC (W) groups. Although 36 DEGs were identified in this comparison, enrichment analysis did not yield robustly significant multi-gene GO terms, indicating that the transition from optimal to saturated moisture elicits specific transcriptomic adjustments rather than broad systemic pathway reprogramming.

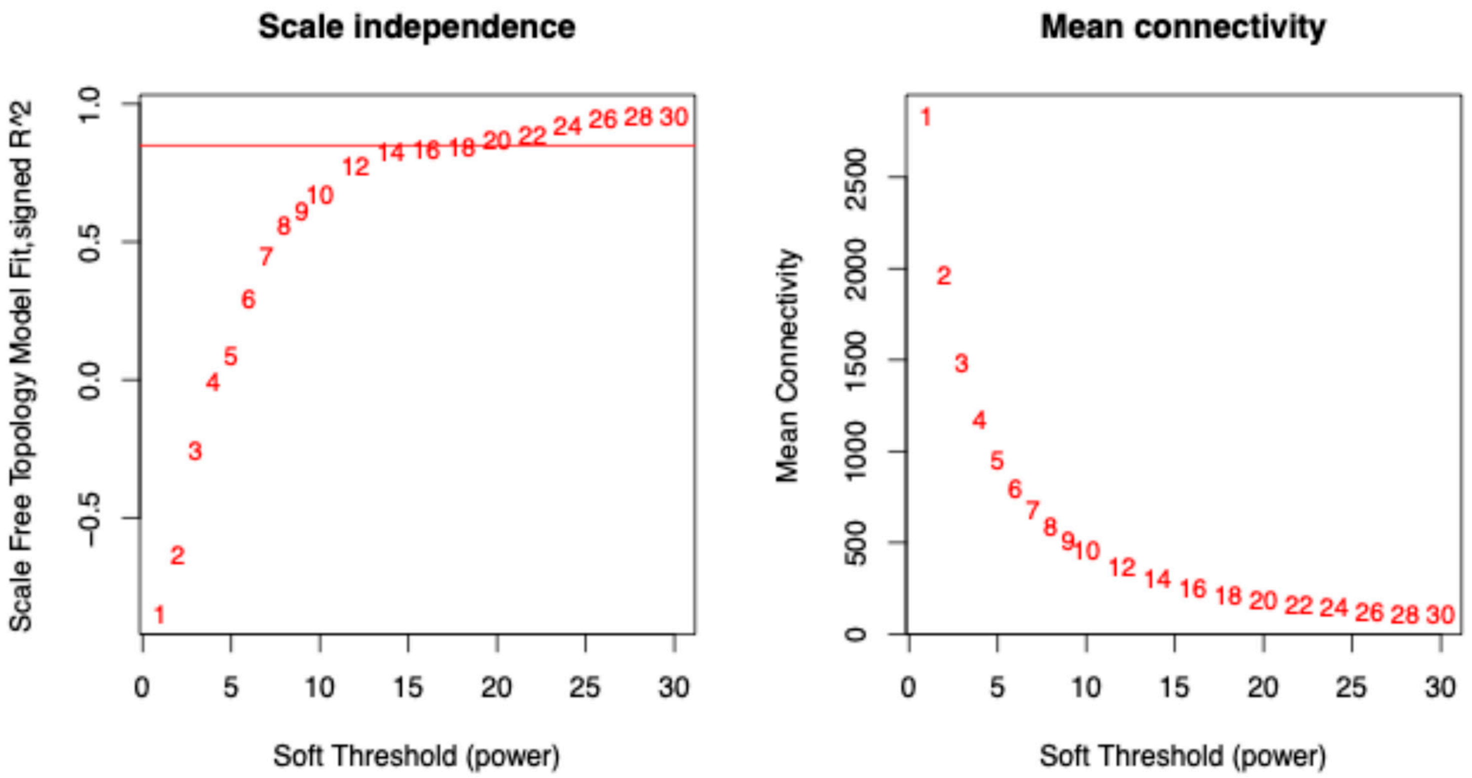

Supplementary Figure S12. Selection of the soft-thresholding power for weighted gene co-expression network analysis (WGCNA). The left panel shows the scale-free topology fit index (R^2) as a function of candidate soft-thresholding powers (1–30); the right panel shows the corresponding mean connectivity. A power of 16 was selected as the lowest value at which R^2 exceeded 0.85 while maintaining adequate network connectivity.

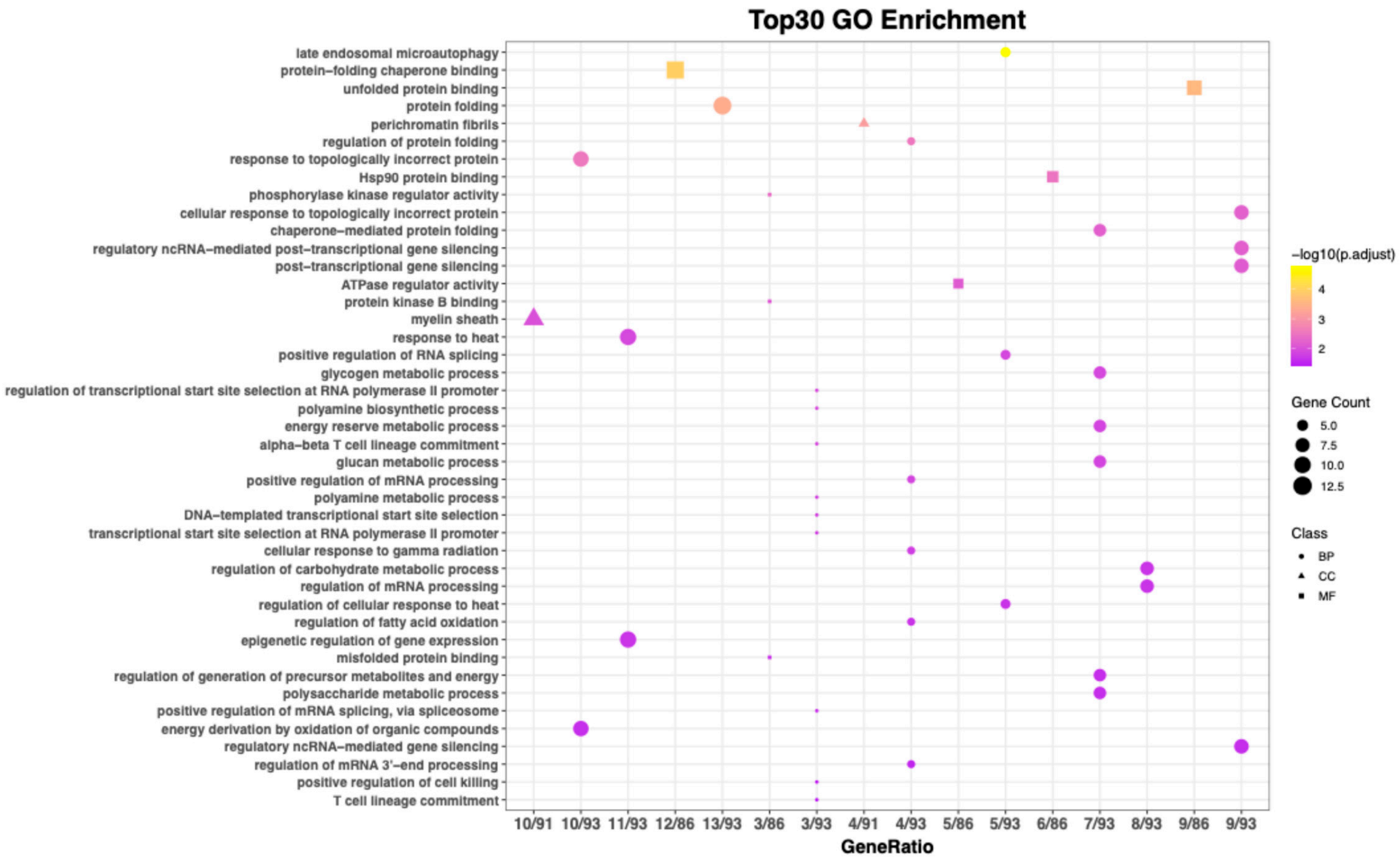

Supplementary Figure S13. Gene Ontology (GO) enrichment analysis of genes within the ME5 (green) module identified by WGCNA. Enriched terms include protein folding, chaperone-mediated protein folding, and late endosomal microautophagy, confirming that the desiccation-correlated module is functionally centered on protein quality control and proteostasis maintenance.

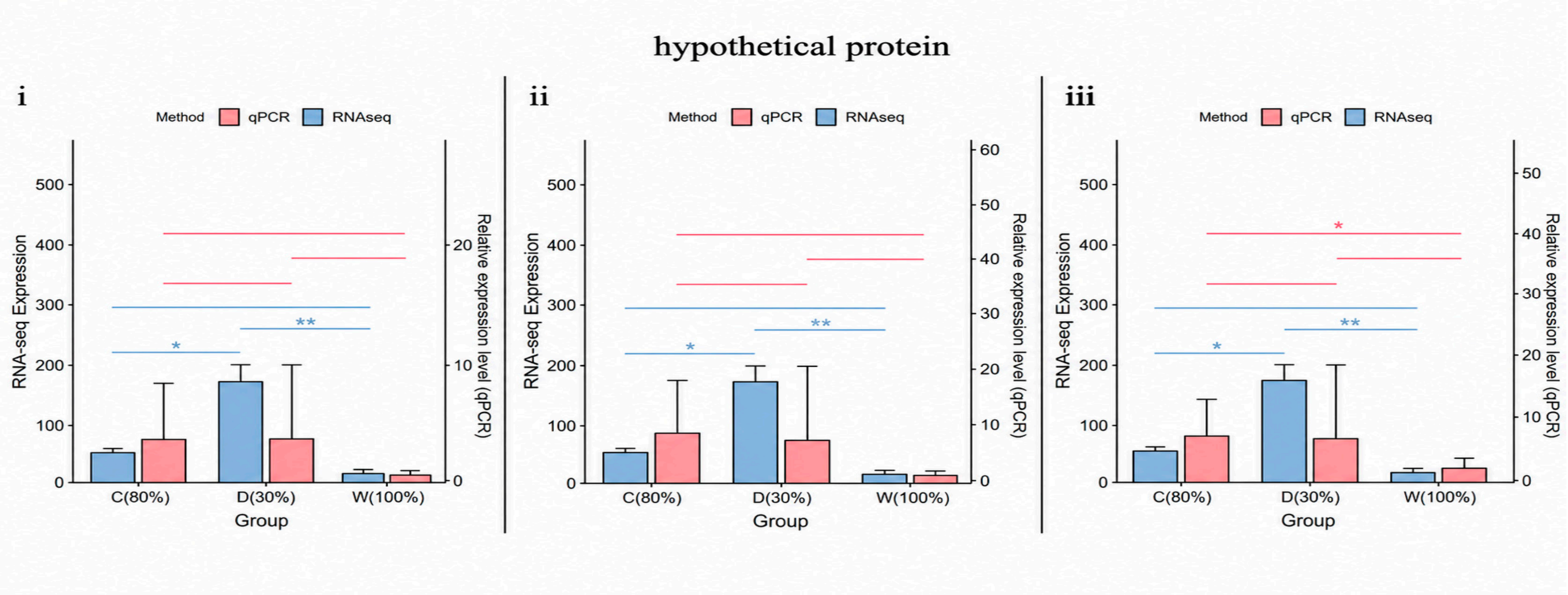

Supplementary Figure S14. qRT-PCR expression profiles of a gene that did not reproduce the RNA-seq expression patterns across the three moisture treatments: dry 30% WHC (D), control 80% WHC (C), water-saturated 100% WHC C (W). Error bars represent standard error of the mean across biological replicates. Within each gene panel, i, ii, and iii denote qRT-PCR normalisation to housekeeping gene: GAPDH, ribosomal protein L11, and ribosomal protein L3, respectively.

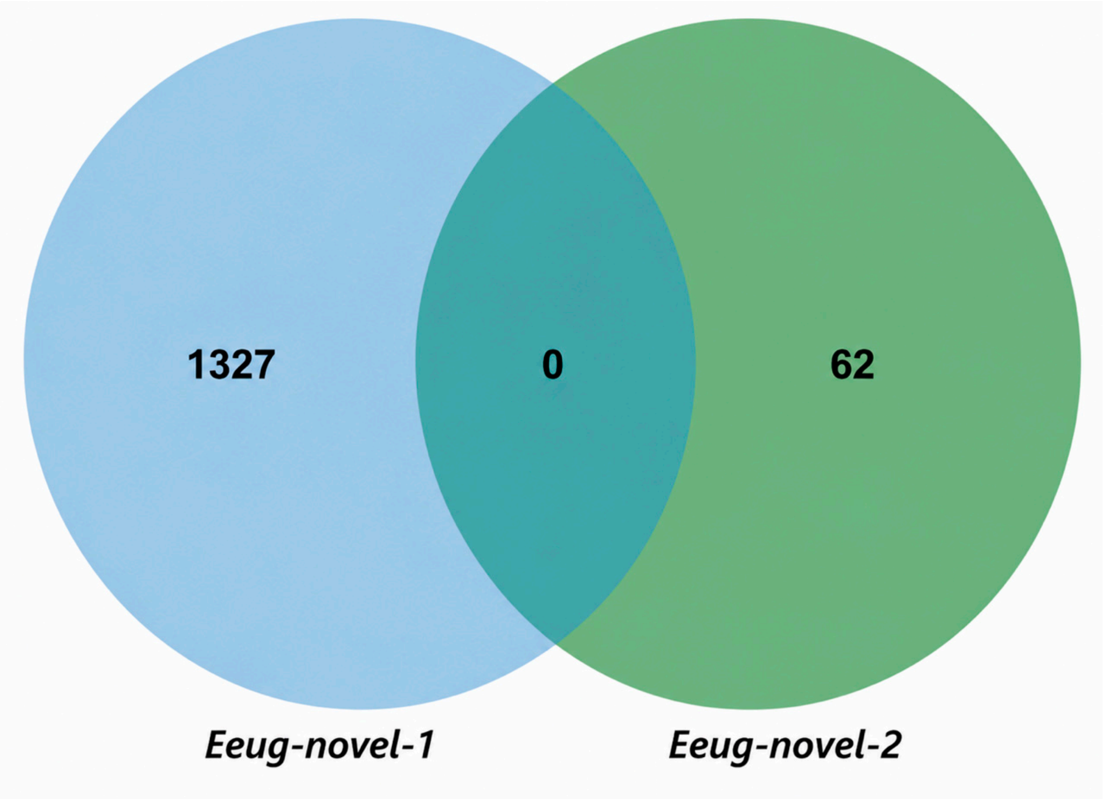

Supplementary Figure S15. Venn diagram of predicted target genes of *Eeug-novel-1* and *Eeug-novel-2* in *E. eugeniae*.

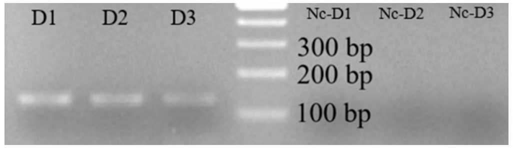

TRINITY_DN989_c7_g1

Supplementary Figure S16. Labeled microRNA pull-down (LAMP) assay results for the interaction between *Eeug-novel-1* and the CHORD-containing protein transcript (D1–D3, three biological replicates; NC, negative controls).

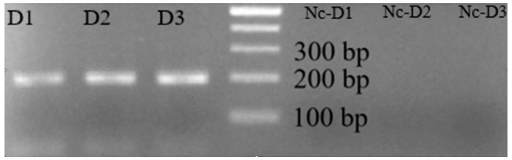

TRINITY_DN17171_c0_g1

Supplementary Figure S17. LAMP assay results for the interaction between *Eeug-novel-1* and Hsp90-like (D1–D3, three biological replicates; NC, negative controls).

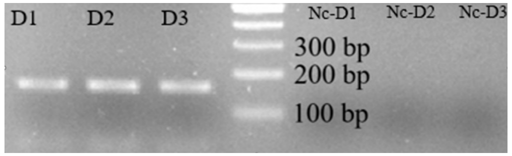

TRINITY_DN3170_c0_g1

Supplementary Figure S18. LAMP assay results for the interaction between *Eeug-novel-1* and Hsp40 *(D1*–D3, three biological replicates; NC, negative controls).

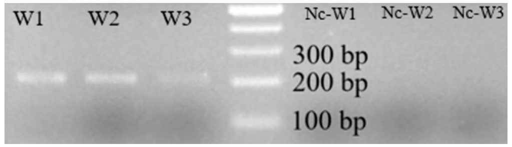

TRINITY_DN1458_c0_g1

Supplementary Figure S19. LAMP assay results for the interaction between *Eeug-novel-2* and the KLF2 transcript (W1–W3, three biological replicates; NC, negative controls).
